# Molecular Detection of *Anaplasma* spp. in Horses From Golestan Province, Northern Iran

**DOI:** 10.1002/vms3.71124

**Published:** 2026-08-06

**Authors:** Abdolrreza Mohammadi Rad, Heidar Rahimi, Emad Changizi, Hamid Staji

**Affiliations:** ^1^ Department of Pathobiology Faculty of Veterinary Medicine Semnan University Semnan Iran

**Keywords:** *Anaplasma*, blood, horse, Iran, PCR, Tick‐borne

## Abstract

**Background:**

Anaplasmosis is a bacterial zoonotic disease that affects both humans and many animals. In horses, anaplasmosis manifests as a seasonal, infectious, and febrile disease caused by bacteria of the genus *Anaplasma*. Since the transmission of this disease is primarily mediated by arthropods such as ticks, it holds significant public health importance.

**Objective:**

The present study aims to investigate the prevalence of anaplasmosis in horses in northern Iran (Golestan Province).

**Methods:**

A total of 350 blood samples were randomly collected from asymptomatic horses between December 2023 and April 2024. Genomic DNA was extracted from whole blood, and conventional PCR targeting a partial fragment of the *16S rRNA* gene was performed for the detection of *Anaplasma* spp.

**Results:**

The prevalence of infection was 15.7% (95% CI: 12.2%–19.8%). Significant variation was observed between geographic regions, with the highest prevalence in Aq Qala (24.3%) and the lowest in Gorgan (7.9%). Age and sex showed no significant effect on infection rates.

**Main Limitations:**

Due to the loss and degradation of DNA samples, sequencing of PCR‐positive samples was not performed, and identification was carried out at the genus level.

**Conclusion:**

These findings demonstrate the detection of *Anaplasma* spp. DNA in horse blood samples from Golestan Province, northern Iran, and highlight the need for further studies on vector identification, species‐level characterization, and the clinical significance of these molecular findings. This study provides baseline molecular epidemiological data on *Anaplasma* spp. in horses in Iran, contributing to future surveillance and control programs.

## Graphical abstract

Using PCR, *Anaplasma* spp. was detected in 15.7% of apparently healthy horses in northern Iran, showing notable differences in prevalence across different regions.

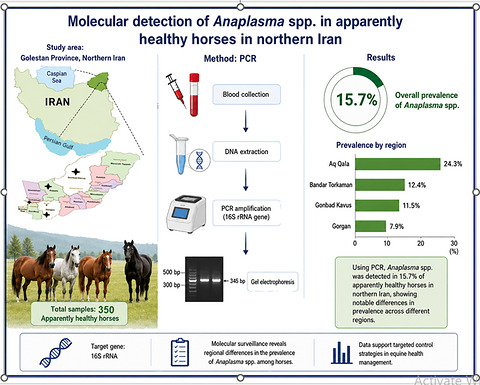

## 1 Introduction

Anaplasma bacteria are considered important arthropod‐borne pathogens and can cause severe illness for humans and animals (Ybañez and Inokuma 2016). This infection has long been recognized as a tick‐borne multi‐system disease affecting several species of wild and domestic mammals, including horses, ruminants, and carnivores (Matei et al. 2019). Anaplasmosis in horses, caused by *Anaplasma* spp., is a seasonal disease of clinical significance that can lead to fever, limb edema, anorexia, depression, jaundice, and hematological abnormalities such as thrombocytopenia and anemia. This disease weakens the horse's immune system, making the animal more susceptible to secondary infections (Bogdan et al. 2024). Since ticks are the primary vectors of this disease, and their distribution is expanding due to climate change, anaplasmosis has become an increasing concern for both veterinary and public health sectors (Matei et al. 2019; Kruppenbacher et al. 2023). Horses play an important economic and cultural role in Iran, especially in Northern provinces such as Golestan, which hosts the country's largest horse population. These animals are extensively used not only for traditional purposes but also increasingly in transportation and equestrian sports (DuBois et al. 2018).

In Iran, several *Anaplasma* species have been reported in livestock, particularly in ruminants, highlighting the widespread distribution of this genus in the region. Five species, including *Anaplasma marginale, Anaplasma phagocytophilum*, *Anaplasma centrale*, *Anaplasma bovis*, and *Anaplasma ovis*, are well recognized in Iranian cattle, sheep, and goats (Noaman 2019, 2020; Khamesipour and Doosti 2022; Noaman and Shayan 2025). These findings indicate that Iran represents an endemic area for *Anaplasma* spp., with diverse species circulating among different host populations (Noaman and Beiranvand 2022; Noaman and Sazmand 2021). Understanding this broader epidemiological context is important for interpreting the occurrence of *Anaplasma* infections in horses and assessing their potential implications.

Despite this, there is limited epidemiological data on equine anaplasmosis in Iran. Investigating the prevalence and risk factors of *Anaplasma* infection in horses in this region is crucial due to its potential epidemiological and public health implications (particularly if zoonotic species such as *A. phagocytophilum* are present), the economic impact on the equine industry, and the broader implications for animal health management (DuBois et al. 2018; Hurtado et al. 2020). The acute phase of the disease can be directly diagnosed by microscopic identification of inclusions in peripheral granulocytes, polymerase chain reaction (PCR), and Giemsa‐stained blood smears. However, in the chronic phase of the disease, PCR is the best diagnostic method. This disease is more common during the warmer seasons due to the higher presence of ticks, and it also shows a greater prevalence in older horses due to increased contact with ticks (Hurtado et al. 2020; Pusterla and Madigan 2013). The biological vectors of this organism include hard ticks, with *Ixodes* being one of the most important. Anaplasmosis weakens the host's immune system, making horses more susceptible to opportunistic or secondary infections caused by viruses, bacteria, and fungi (Pusterla and Madigan 2013). The number of equine anaplasmosis cases in the United States increased from 348 cases in 2000 to 6,729 cases in 2021, showing a growth rate faster than other Tick‐borne diseases (MacQueen and Centellas 2022). Ninety‐nine thousand nine hundred fifty‐one cases of human granulocytic anaplasmosis (HGA) were reported in the United States between 2000 and 2021. Due to its zoonotic potential, prompt and precise diagnosis plays a critical role in managing and preventing the spread of anaplasmosis. Molecular methods are reliable for diagnosing equine anaplasmosis and identifying subclinical infections (Athanasiou et al. 2023). Therefore, in the present study, the epidemiology of this disease in asymptomatic equines in northern Iran was investigated by detecting *Anaplasma* spp. DNA in blood using PCR, and associated risk factors including geographic location, age, and sex were also evaluated.

## 2 Materials and Methods

### 2.1 Study Area

**FIGURE 1:**
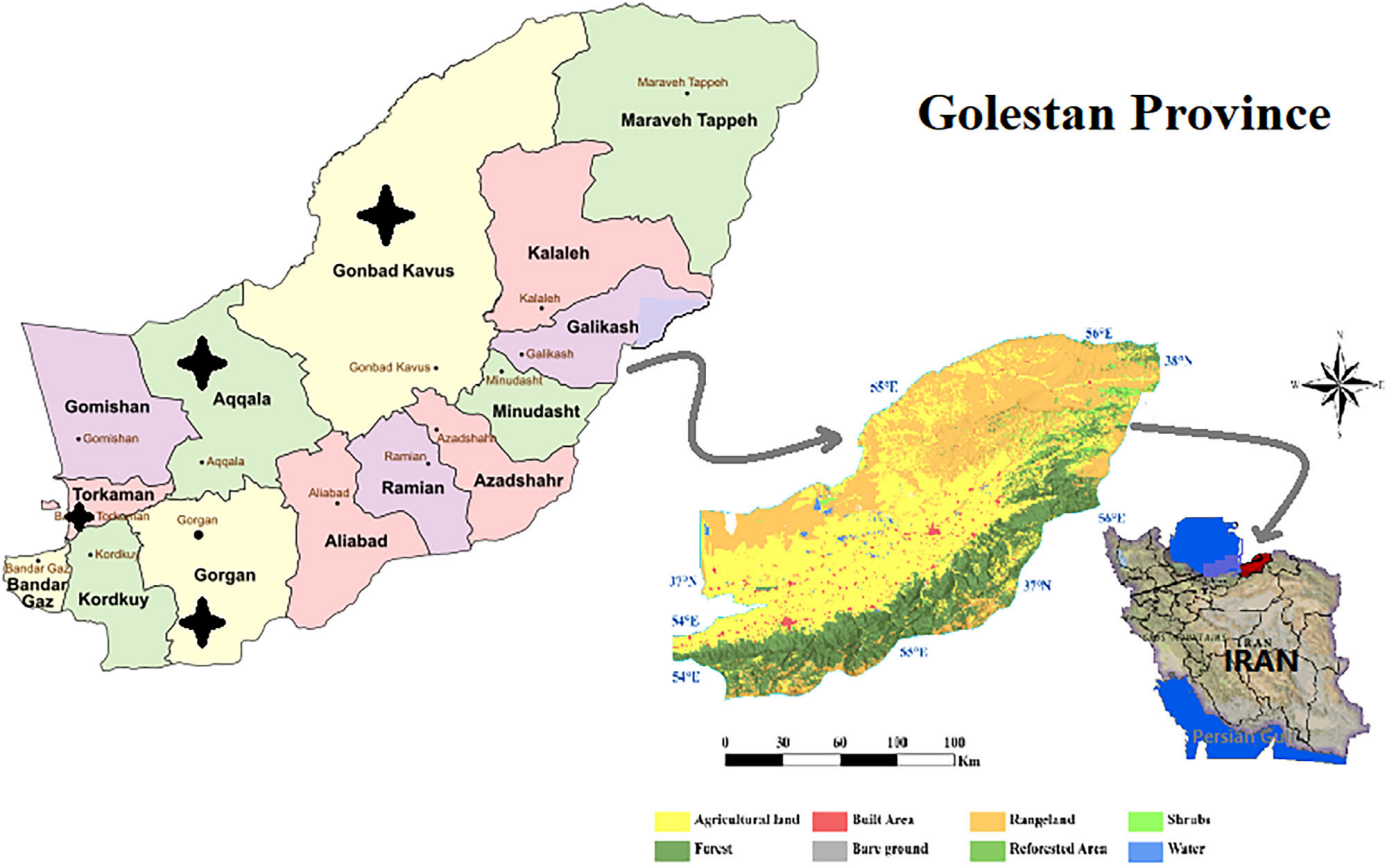
Map of Iran showing the geographic location of the study areas mentioned in article. The areas marked with a star are the studied regions.

The present study was conducted in northern Iran (Golestan Province), which has the highest number of horses in the country. This province, with a temperate climate, is located in the northern part of the country, between the latitudes 36°30′ to 38°8′ north and longitudes 53°57′ to 56°22′ east of the Greenwich Meridian. The area of the province is 22,022 square kilometers, accounting for approximately 1.33% of the total area of the country. Around 70% of the area of Golestan Province is covered by natural resources Figure 1.

### 2.2 Sampling

From December 2023 to April 2024, a total of 350 blood samples were collected from the jugular vein of horses in Golestan Province (districts of Gorgan 38 heads [10.8%], Bandar Torkaman 145 heads [41.4%], Gonbad Kavus 52 heads [14.8%], and Aq‐Qala 115 heads [32.8%]). The collected samples were categorized by sex (212 females (60.6%) and 138 males (39.4%)) and divided into three age groups: less than 3 years (*n* = 224), between 3 and 5 years (*n* = 96), and more than 5 years (*n* = 30). Sampling was conducted in collaboration with the Golestan Provincial Veterinary Organization during the provincial glanders surveillance program. At the time of sample collection, no visible tick infestation was observed on any of the sampled horses. During sampling, blood was collected into both EDTA and plain tubes. EDTA‐anticoagulated blood was used for the molecular detection of *Anaplasma* spp. in the present study. The four study districts were selected because they contain the highest equine populations in Golestan Province. A formal sample size calculation was not performed because no previous molecular epidemiological data on *Anaplasma* spp. in horses were available for the study area. Therefore, the sample size was determined based on the surveillance program design, horse availability during the study period, and logistical feasibility. Horses were randomly selected during field sampling within the selected districts to ensure broad geographic coverage. Blood samples were stored at −20°C until DNA extraction.

### 2.3 DNA Extraction and PCR Amplification

A volume of 200 µL from each blood sample was added to a sterile 1.5 mL microtube. DNA was then extracted from the blood samples using the Smobio, DNA extraction kit (Smobio, Taiwan) according to the manufacturer's instructions. The purity and concentration of extracted DNA were evaluated using a NanoDrop ND‐1000 spectrophotometer (Thermo Scientific, USA), and samples with an A260/280 ratio between 1.8 and 2.0 were considered suitable for PCR analysis. All DNA samples were stored at −20°C until further processing. Conventional PCR was performed to detect *Anaplasma* spp. by amplifying a partial fragment of the *16S rRNA* gene using previously published primers (Parola and Raoult 2001). As this assay detects *Anaplasma* at the genus level, it does not permit species‐level identification. Therefore, further sequencing or species‐specific PCR would be required to identify the infecting *Anaplasma* species. The PCR conditions included an initial denaturation step at 95°C for 5 min, followed by 35 cycles of denaturation at 94°C for 30 s, annealing at 58°C for 30 s, extension at 72°C for 45 s, and a final extension step at 72°C for 10 min. The amplified product size was 345 base pairs (Parola and Raoult 2001).

PCR amplification was performed using the PCR Master Kit (Ampliqon Taq DNA Polymerase Master Mix RED 1.25 mL, Ampliqon, Denmark) with a 25 µL reaction mixture containing 12.5 µL of 2X Master Mix, 0.5 µL of forward and reverse primers, 5 µL of DNA, and 6 µL of DNA‐free water. The positive control DNA was obtained from a previously confirmed *Anaplasma*‐positive sample used in a study conducted at Urmia University (Esmaeilnejad et al. 2026). For the negative control, sterile deionized distilled water was used. The PCR products were then separated on a 1.5% agarose gel (Merk, Germany), containing 2 µg/mL safe stain (Cinna Gene, Tehran, Iran). Electrophoresis was performed in 0.5x Tris/borate/EDTA (TBE) buffer at 90 volts for 1 h. A 100 base pair DNA ladder (Smobio, Taiwan) was used as a molecular weight marker.

### 2.4 Statistical Analysis

Statistical analyses were performed using SPSS software version 22 (SPSS Inc., Chicago, IL, USA). Associations between *Anaplasma* spp. PCR positivity and categorical variables (sex, age group, and geographic region) were evaluated using Pearson's chi‐square test. Exact *p*‐values, chi‐square statistics (χ^2^), and degrees of freedom (df) were reported. The assumptions of the chi‐square test were assessed before analysis, and exact tests were considered where appropriate for variables with small expected cell counts. A *P*‐value < 0.05 was considered statistically significant.

## 3 Results

**CHART 1:**
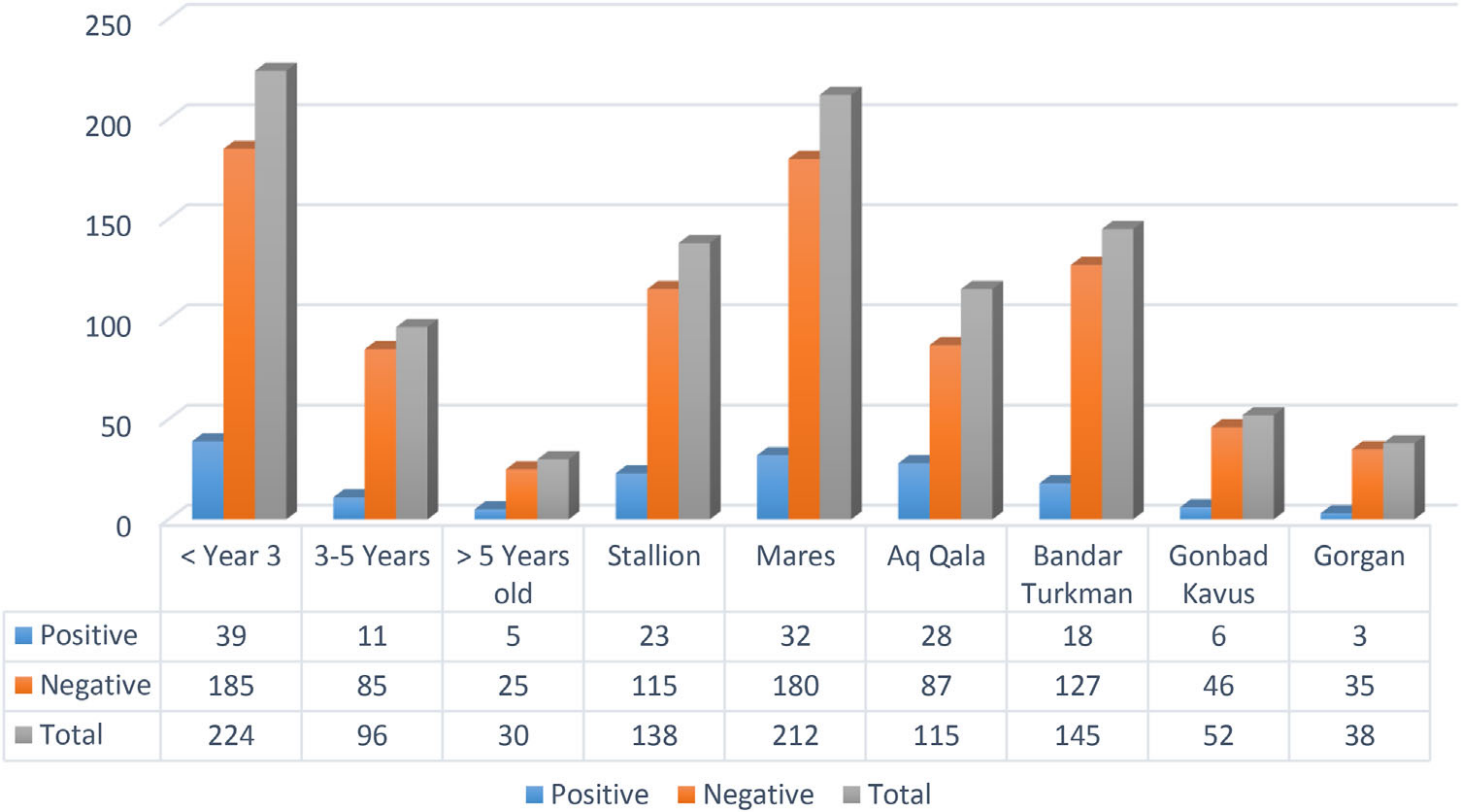
Prevalence of anaplasmosis in horses based on age, gender, and geographic region.

**FIGURE 2:**
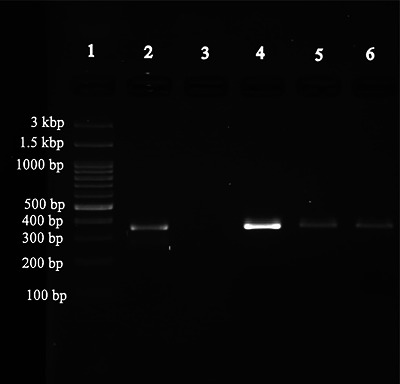
Agarose gel image of amplified fragment for the detection of *Anaplasma* spp. Lane 1: Standard DNA marker. Lane 2: Positive control (345bp). Lane 3: Negative control. Lanes 4–6: Positive samples with *Anaplasma* spp. DNA.

In this study, out of 350 horse blood samples, 55 samples tested positive for *Anaplasma* spp. DNA, indicating a prevalence of 15.7% (*P* < 0.05, 95% CI: 12.2%–19.8%). Among the positive cases, 32 horses (15%) were female and 23 horses (16.6%) were male. There was no statistically significant association between gender and infection with anaplasmosis (χ^2^ = 0.060, df = 1, *P* = 0.807). The infection rate in horses younger than 3 years was 17.4% (*P* < 0.05, 95% CI: 13.0%–22.9%), which was higher than that in other age groups. However, no significant relationship was observed between age and anaplasmosis infection (χ^2^ = 1.820, df = 2, *P* = 0.403). Horses from the Aq Qala region showed the highest prevalence at 24.3% (*P* < 0.05, 95% CI: 17.4%–32.9%), whereas horses from the Gorgan region exhibited the lowest prevalence at 7.9% (*P* < 0.05, 95% CI: 2.7%–20.7%). A statistically significant association was found between geographic location and the prevalence of anaplasmosis (χ^2^ = 10.103, df = 3, *P* = 0.018). The statistical analysis of the association between *Anaplasma* spp. PCR positivity and the evaluated variables (sex, age group, and geographic region) is presented in Table 1 and Chart 1. An agarose gel image of the amplified 345 bp fragment of the *Anaplasma*‐specific *16S rRNA* gene is shown in Figure 2.

**TABLE 1:** Statistical analysis of data for results *Anaplasma* spp. DNA detection (sex, age and region).

Variable	Category	Frequency	PCR‐positive	95 % CI	χ^2^ (df(	*P*‐value
Total sample	−	350	55 (15.7 %)	12.2–19.8	−	−
Sex	Stallion	138	23 (16.6 %)	11.3–23.7	0.060(1)	0.807
	Mares	212	32 (15.0 %)	10.9–20.5		
Age group	<3 years	224	39 (17.4 %)	13.0–22.9	1.820(2)	0.403
	3–5 years	96	11 (11.4 %)	6.52–19.3		
	>5 years	30	5 (16.6 %)	7.34–33.5		
Region	Aq Qala	115	28 (24.3 %)	17.4–32.9	10.103(3)	0.018
	Bandar Torkaman	145	18 (12.4 %)	8.0–18.7		
	Gonbad Kavus	52	6 (11.5 %)	5.4–22.9		
	Gorgan	38	3 (7.9 %)	2.7–20.7		

Abbreviations: CI, 95% confidence interval; df, degrees of freedom; χ^2^, Pearson's chi‐square test statistic.

## 4 Discussion

Global warming has significantly affected the distribution of blood‐feeding arthropods, including ticks (Hussain et al. 2022). As a result, tick‐borne diseases have become significant concerns for human and animal health. One of the most important tick‐borne diseases is anaplasmosis, which is associated with significant infection rates and mortality (Dumler et al. 2005). Recent studies have identified whole blood as the most suitable sample type and PCR as the most reliable method for detecting anaplasmosis in horses (Seo et al. 2023; Schäfer et al. 2022). Accordingly, in the present study, by analyzing 350 blood samples of horses collected from northern Iran, regions with the highest horse populations, the prevalence of *Anaplasma* spp. was found to be 15.7%. This result indicates that horses in Iran exhibit a high level of infection with *Anaplasma* spp. In Iran, previous molecular studies conducted in horses have mainly reported Anaplasma‐related infections at the species level rather than providing an overall genus‐level prevalence. For example, Akbari et al. (2024) reported the presence of *A. phagocytophilum* (2%–4%) and *Anaplasma ovis* (1%–3%) in horses from northwestern Iran. Similarly, Shams et al. (2026) identified members of the Anaplasmataceae family, including *Ehrlichia canis* (4%) and *Anaplasma ovis* (1.5%) in Turkmen horses from northern Iran. Despite differences in study design and diagnostic approaches, these findings indicate exposure of equine populations in different regions of Iran to Anaplasma‐related agents and are generally consistent with the results of the present study. When comparing the present findings with international reports, a similar prevalence has been observed in several countries; a prevalence similar to that found in horses in Germany 15.2% (Schäfer et al. 2022), Pakistan 10.67% (Saleem et al. 2018) and Iraq 13.1% (Alani and Yousif 2023). In contrast, a lower prevalence has been reported in countries such as South Korea, where the infection rate among horses is 3.5% (Seo et al. 2023), and 0.2% (Seo et al. 2018). This lower prevalence in South Korea may be influenced by several factors, including differences in climate, tick vector species, and their distribution patterns. South Korea's temperate climate and seasonal variations may affect the activity and population density of tick species capable of transmitting *Anaplasma* spp. Additionally, differences in horse management practices, habitat types, and sampling periods between studies could contribute to the variability in prevalence rates. However, due to limited data on tick ecology and environmental conditions in the regions studied, a comprehensive comparison remains challenging. Future studies focusing on vector identification and environmental factors are needed to clarify these discrepancies.

Studies conducted in Iran indicate that the prevalence of *Anaplasma* spp. infection in ticks is relatively high. For instance, in a study by Hosseini‐Vasoukolaei et al. (2014) conducted in northern Iran, 49.5% of ticks were infected. In another study by Hosseini‐Chegeni et al. (2020), in western and northwestern Iran, the prevalence was 2.3%, while Ranjbar et al. (2020) reported a 6.6% infection rate in southeastern Iran. Given that the horses examined in the present study were exclusively from northern Iran, the findings are consistent with those of Hosseini‐Vasoukolaei et al. (2014), indicating a high prevalence of anaplasmosis in ticks and horses in this region. Hard ticks, the primary vectors of anaplasmosis, are most active during spring and summer, when acute infections are more commonly detected. In the present study, all samples were collected during winter and early spring from clinically healthy horses; therefore, the detected infections may represent chronic cases, although this cannot be confirmed without longitudinal or serological data.

The results clearly indicate that the prevalence of tick‐borne diseases, such as anaplasmosis investigated in this study, is influenced by climatic and regional factors. The study demonstrated a significant association between the prevalence of anaplasmosis in horses and their habitat. Horses from Aq Qala showed the highest infection rate at 24.3%, while those from Gorgan had the lowest at 7.9%. Aq Qala has the highest population of ruminants among the cities in Golestan Province and features a warmer climate compared to Gorgan. Additionally, most of Aq Qala consists of extensive pastureland and agricultural land, whereas Gorgan is predominantly forested. These environmental differences may contribute to a higher tick population in Aq Qala compared to Gorgan and other areas of the province, which could explain the higher prevalence of anaplasmosis in horses from Aq Qala observed in this study. It should also be noted that the relatively small sample size in certain geographical areas, particularly Gorgan, resulted in wide confidence intervals, which may reduce the precision of prevalence estimates and should be considered when interpreting these regional differences. However, we acknowledge that our study lacked specific data regarding the tick species and their distribution in this region. We recommend that future research focus on identifying tick species and mapping their distribution to better understand their role in the transmission of Anaplasma in this area.

Although *A. phagocytophilum* is considered the main *Anaplasma* species infecting horses and is known to have zoonotic potential, species‐level identification was not performed in the present study due to the lack of sequencing data. Therefore, the detected infections should be interpreted as *Anaplasma* spp. However, previous studies conducted in neighboring countries such as Pakistan (Saleem et al. 2018) and Iraq (Alani and Yousif 2023), as well as in other regions including Germany (Schäfer et al. 2022), South Korea (Seo et al. 2018), and the northeastern United States (Chien et al. 2023), have predominantly reported *A. phagocytophilum* in horses. Based on these findings, it may be hypothesized that *A. phagocytophilum* could be present in the study area; however, this assumption requires confirmation through sequencing and should be interpreted with caution.

The present study demonstrated no statistically significant difference in the prevalence of anaplasmosis between male and female horses, indicating that gender does not play a role in the occurrence of the disease. The prevalence rates in male and female horses were 16.6% and 15%, respectively. In the current study, horses aged between 3 and 5 years showed a lower prevalence of anaplasmosis than other age groups. However, statistical analysis revealed no significant association between age and anaplasmosis prevalence. Consistent with the findings of Razzaq et al. (2015), the present study showed that animal‐related factors such as age, gender, and species were not significantly associated with anaplasmosis in horses. However, it should be noted that the relatively small sample size in some subgroups, particularly horses older than 5 years, resulted in wide confidence intervals, which may affect the precision of prevalence estimates in these groups and should be considered when interpreting the results.

Although the transmission of anaplasmosis is primarily associated with tick activity, the presence of *Anaplasma* spp. in countries from which horses are imported into Iran, such as Turkey and the United Arab Emirates, may provide relevant epidemiological context. In Turkey, which shares a border with Iran, a prevalence rate of 6.4% in horses has been reported (Oğuz 2021). In the United Arab Emirates, although data on horses are lacking, a prevalence of 11.8% has been reported in camels (El Tigani‐Asil et al. 2021). These reports indicate that *Anaplasma* spp. circulate in the broader region. However, based on the data from the present study, no direct inference can be made regarding the role of horse importation in the epidemiology of anaplasmosis in Iran. The occurrence of the disease is more plausibly associated with local environmental and climatic factors.

Overall, studies have shown that anaplasmosis is highly prevalent among animals in Iran. A systematic review by Soosaraei et al. (2020) reported a prevalence rate of 34% among ruminants in the country. Additionally, Hamidinejat et al. (2019) found a prevalence of 57.2% in rural dogs in Iran. The present study demonstrates a lower prevalence of anaplasmosis in horses than in ruminants and dogs (data on cats in Iran are not yet available). This lower prevalence in horses may be attributed to better management and husbandry practices and a reduced likelihood of contact with vectors such as ticks.

## 5 Limitations

This study has several limitations that should be considered when interpreting the findings. Species‐level identification was not possible because PCR‐positive samples could not be sequenced due to degradation of the stored DNA. Therefore, the results should be interpreted as genus‐level detection of *Anaplasma* spp. A formal sample size calculation was not performed because sampling was conducted within the provincial glanders surveillance program. In addition, information on horse management, animal movement, acaricide treatment, and farm‐level characteristics was not available. Ticks were not collected or examined; therefore, no data were available on tick species, abundance, or *Anaplasma* infection in local tick populations. Although no visible tick infestation was observed during sampling, future studies should include species identification, comprehensive epidemiological data, and integrated tick surveillance to better understand the epidemiology of *Anaplasma* spp. in horses.

## 6 Conclusion

This study demonstrated the detection of *Anaplasma* spp. DNA in horses from Golestan Province, northern Iran, with significant geographic variation among the studied districts. These findings provide baseline molecular epidemiological data on the occurrence of *Anaplasma* spp. in horses in this region. Further studies, including species identification by sequencing or species‐specific PCR, vector investigations, and longitudinal epidemiological surveys, are required to better understand the epidemiology, veterinary significance, and potential public health relevance of *Anaplasma* spp. in Iran.

## 7 Clinical Relevance

This study demonstrated the detection of *Anaplasma* spp. DNA in 15.7% of asymptomatic horses in northern Iran, with significant geographic variation among the studied regions. These findings indicate that *Anaplasma* spp. circulate in the equine population of the study area and support the importance of continued molecular surveillance and vector monitoring in endemic regions. Further studies, including species identification, vector investigations, and longitudinal epidemiological surveys, are required to clarify the epidemiological role of horses and to assess the potential veterinary and public health significance of the detected *Anaplasma* spp., particularly if zoonotic species such as *A. phagocytophilum* are present

## Author Contributions

**Abdolrreza Mohammadi Rad**: methodology, formal analysis. **Heidar Rahimi**: methodology, formal analysis, project administration, investigation, software, writing – review & editing, writing – original draft. **Emad Changizi**: investigation, conceptualization. **Hamid Staji**: investigation, conceptualization.

## Funding

This study was supported by Semnan University (Iran).

## Ethics Statement

Ethical approval for this study was obtained from the Animal Ethics Committee of the Faculty of Veterinary Medicine, University of Semnan (Approval ID: IR.SU.REC.1404.09). Blood samples from horses were collected by the Veterinary Organization of Golestan Province as part of routine diagnostic procedures. No additional harm or intervention was performed on the animals specifically for this research.

## Conflicts of Interest

The authors declare no conflicts of interest.

## Data Availability

All data generated or analyzed during this study are included in this published article. Additional data are available from the corresponding author upon reasonable request.
